# Ecological Composite Materials Based on Polylactide (PLA) and Organic Fillers: Coffee Grounds and Hen Eggshells Produced by the FDM Method: Mechanical, Thermal Properties, Stress Relaxation and Creep

**DOI:** 10.3390/ma18214918

**Published:** 2025-10-28

**Authors:** Anna Gaweł, Kinga Setlak, Damian Szubartowski, Dariusz Mierzwiński, Aneta Liber-Kneć

**Affiliations:** 1 Department of Production Engineering, Faculty of Mechanical Engineering, Cracow University of Technology, 31-864 Kraków, Poland; 2 Department of Materials Engineering, Faculty of Materials Engineering and Physics, Cracow University of Technology, 31-155 Kraków, Poland; kinga.setlak@pk.edu.pl (K.S.); dariusz.mierzwinski@pk.edu.pl (D.M.); 3 Interdisciplinary Center of Circular Economy, Cracow University of Technology, 31-155 Kraków, Poland; aneta.liber@pk.edu.pl; 4 Department of Applied Mechanics and Biomechanics, Faculty of Mechanical Engineering, Cracow University of Technology, 31-864 Kraków, Poland; damian.szubartowski@pk.edu.pl

**Keywords:** polylactide, organic fillers, coffee grounds, eggshells, stress relaxation, creep, Zener model

## Abstract

**Highlights:**

**What are the main findings?**

- The use of organic substances in the production process of polymer composites can be recommended;
- A deterioration in the strength properties of the produced composites can be achieved;
- A reduction in the amount of environmental waste can be achieved.

**What are the implications of the main findings?**

- Lower production costs, and reduced carbon dioxide emissions;
- Increased potential for environmental degradation;
- The use of waste for production can be recommended.

**Abstract:**

In this article, an ecological composite based on a neat polylactide with 50 and 75% degrees of coffee particles and eggshells as an infill and organic filler, was developed. It has been shown that the content of fillers used reduced the mechanical properties, increasing the possibility of environmental degradation and accelerating the biodegradation process. During the additive production of polylactide with 10% of coffee grounds as a filler, it was possible to reduce the additive manufacturing temperature, which reduced the process time, energy costs, carbon dioxide emissions and the amount of polymer that may affect the environment. The structure of polylactide enriched with hen eggshells is characterized by roan and irregular shapes, which can cause a high tendency to form a concentration of cracks in these areas. Based on the results obtained from the stress relaxation test, the Zener model was used to describe a creep model of the produced ecological composites. The polymer composition of coffee grounds and eggshells shows a tendency to creep faster than pure polylactide and with different degrees of infill. Voids reduce the strength of composite materials, which increases the creep potential of samples with incomplete degrees of infill.

## 1. Introduction

Nowadays, the most frequently used plastics are polyethylene (PE), polyvinyl chloride (PVC), polypropylene (PP) and polystyrene (PS), which are produced from crude oil and its derivatives [1,2]. Massive consumption of crude oil worldwide is leading to a depletion of this resource [1]. Additionally, these materials are non-biodegradable and incompatible with the human body [2,3], which has led many researchers to try to introduce solutions that are friendly to the environment [4]. Such materials include, polylactic acid (PLA), polylactic-co-glycolic acid (PLGA), polyethylene glycol (PEG), polyhydroxybutane (PHB), and polycaprolactone (PCL) [5,6], among others.

Among the polymer materials mentioned, polylactide is an increasingly popular material used in the industry [7]. It is a thermoplastic polyester obtained from lactic acid monomer in the following processes: ring-opening polymerization (ROP), poly-condensation, azeotropic dehydration and enzymatic polymerization. Obtaining PLA with a high molecular weight requires high temperatures ranging from 180 to 200 [°C] and a low pressure equal to 5 [mm Hg]. The most commonly used methods are ROP and lactic acid polycondensation, where lactic acid monomer is produced by extracting sugar or starch from natural sources such as corn, sugar beet, starch or soy protein through fermentation or petrochemical processes [8]. By changing the arrangement of the L-lactic acid (L-LLA) and D-lactic acid (D-LA) monomers, the chemical structure of polylactide can be modified using appropriate catalysts and polymerization reaction conditions. Polymeric materials such as PLA are characterized by amorphous and crystalline structures, which can be obtained by modifying the stereochemical composition of the monomers. The final properties of polymer produced depend on the chemical composition of the monomer present in the polymer [9]. Due to the occurrence of CH_3_ in the chemical structure, PLA has hydrophobic properties [5,6]. Although PLA is hydrophobic, it can exhibit increasing hydrophilicity over time or under conditions like aging, hydrolysis, and oxidation. During natural aging, the hydrolytic degradation of the ester bonds leads to the formation of carboxyl and hydroxyl end groups, which enhances the material’s affinity to water. Additionally, surface oxidation and morphological changes such as chain scission or microcracking can expose more polar groups (-OH, -COOH), further promoting water absorption and wettability. Consequently, aged PLA tends to show lower water contact angles and higher moisture uptake compared to freshly processed PLA environmental behavior [7,10].

Good processing properties and a number of ecological advantages make polylactide the most commonly used material for 3D printing. Fernandes et al. showed that a higher degree of infill of the manufactured elements during the 3D printing process results in better properties of tested material [11]. Reducing the degree of infill increases the amount of empty spaces, which generates decohesion and decreases the elasticity of polymer [12]. The type of infill also has a direct influence on the physical and mechanical properties [13]. It is possible to orient individual filament threads created during 3D printing [14]. Designing the material in a honeycomb shape makes the material more durable during the destruction process [15]. This is related to the fracture path, which in the case of a regular, cylindrical structure will slow down at the bond boundaries. A spherical region means there are no corners where according to the principles of fracture mechanics, the decohesion process begins [16,17].

Contemporary ecological trends and widespread consumerism have led to the increasingly frequent use of composite materials made of polylactide and organic additives [18]. Commonly used additives include wood [19], cellulose fibers [20], bamboo fibers [21] starch [22], coffee [23] and hen eggshells [24]. Coffee is the second most consumed beverage in the world, which results in large amounts of waste from this raw material. Using coffee as a filler in the production of composite materials reduces the amount of polymer material used [25]. Yu et al. produced a polylactide composite containing 1, 3, 5, and 7% coffee particles. They showed that the best mechanical properties were found in samples with a 3% filler, while the highest degree of crystallinity was proven in samples with a 7% filler [23]. Carpintero et al. used egg yolk as a plasticizer to improve the processability of polylactide [26]. Hanumantharaju et al. prepared a composite of polylactide with hen eggshells with 10%, 15%, and 30% filler produced by injection molding. They demonstrated that the sample containing 10% of eggshells had the best biodegradability [24]. Yiga et al. showed that adding rice husks to the PLA matrix causes an increase in the melting point. The addition of rice husks inhibited the neat PLA pyrolysis process [27]. Pereira et al. studied PLA filled with up to 20% rice husks. The best printability was obtained with 5% HR content and it was the value characterized by the lowest decrease in mechanical properties [28]. In this article, Balart et al. added 10–40% hazelnut shells to the polylactide matrix. The degree of crystallinity increased with increasing HSF, mainly due to the nucleating effect of lignocellulosic particles [29]. Ochi et al. investigated the effect of molding temperature and fiber content on the flexural properties of bamboo fiber-reinforced composites. The flexural strength of this composite increased with increasing fiber content, up to 70%. Producing bio-composites at a temperature of 180 °C causes a decrease in the flexural strength of the composites [30].

Polylactide is widely used in tissue engineering due to its antibacterial properties and biocompatibility. Alam et al. treated copper, bronze, and silver particles with acetic acid to obtain antibacterial, thin, and porous scaffold surfaces [31]. Baran et al. described a PLA–halloysite nanotube (HNT) composite filled with zinc nanoparticles (PLA + HNT + Zn), obtaining a hydrophobic surface, and covered it with two layers of Fetal Bovine Serum (FBS) on the sides and one layer of NaOH in the center. Additionally, they coated the outer layer with gentamicin, which was intended to protect against bacterial infections. This solution increased hydrophilicity and cell adhesion [32].

Tüfekci et al. described the effect of printing angles of 0, 45, and 90° on the stress relaxation of polylactide. They found that samples made at an angle of 45° comprised the most elastic group. Samples made at an angle of 90° had the lowest strength properties during tensile and bending tests [33]. Although many organic additives have been tested for their mechanical and thermal properties [24,25,34,35], no data were found in the literature relating to the stress relaxation of PLA with organic fillers, forming the basis of the following studies. Such tests are important for determining long-term mechanical properties, modeling material behavior under constant load, optimizing composition, and predicting service life and degradation over time.

## 2. Materials and Methods

### 2.1. Materials and Composite Preparation

The matrix for the produced composites was polylactide, obtained from FKuR Kunststoff GmbH (Willich, Germany) under the trade name Bio-Flex^®^ 3D Clear. Two groups of composites were produced: polylactide as a matrix, with 10% of coffee particles and 10% of hen eggshell particles. MK Cafe Premium ground coffee was obtained as a waste from the brewing process. Eggshells and coffee were ground through a sieve. The resulting composites, in the appropriate proportions, and pure polylactide were extruded using 3DEVO Composer 350/450 (Holandia, 3devo Company, Utrecht, The Netherlands), a single-screw extruder to produce a filament with a diameter of 1.75 mm. Dog bone-shaped samples were produced for strength tests in accordance with the EN ISO 3167 standard [36] for plastics on a 3DGenceOne printer using the fused deposition modeling (FDM) method. The basic parameters used during sample production are presented in Table 1. The thickness of the 3D printing line directly affects the strength properties of the polymers and composites. The thinner single print line, the higher the strength properties. This is related to the reduction in voids in the structure of the printed material. The process of 3D printing is inherently porous due to the interconnection of individual, small-filament strands, compared to other manufacturing methods, such as injection molding. Increasing voids also increases hydrophilicity, a finding confirmed in the literature [37]. Differential scanning calorimetry (DSC) was performed on a NETZSCH DSC 3500 Sirius (Selb, Germany) apparatus. The specimens for the DSC tests were prepared in the same way as the samples for mechanical characterization were. All the samples, weighing about 10 mg each, were cut from the prepared specimens. The analyses were performed under a nitrogen flow (20 mL/min) at a temperature from 20 °C to 200 °C. The rate of heating and cooling was 10 K/min. The heat–cold–heat approach was used to remove the thermal background.

Due to the specific nature of 3D printing, it can be expected that the strength properties of composite materials will decrease significantly with increasing nozzle diameter. In this research, the highest possible strength with the most optimized parameters was possible to obtain with a diameter of 0.6 mm.

As for the selected fillers, coffee particles and eggshells, used as biodegradable waste materials, have positive impacts on the reduction in carbon dioxide emissions and in production costs, and accelerate the environmental degradation process. The composites produced constitute a broad group of HGPCs. Based on this, the visual aspects of the produced materials was also improved, expanding their range of applications. The chemical compositions are presented in Table 2.

The samples labeled with the letters “Cr” were crystallized at 70 °C for 1 h and subsequently subjected to slow air cooling for 24 h. This process was conducted after 3D printing in order to increase the strength properties related to the formation of a crystalline structure. The degree of crystallinity increases after crystallization in polylactide, which results in increased material strength. This work focuses on comparing the thermal properties of pure polylactide and eco-friendly composites. Samples designated as non-crystallized are simply printed and tested.

In this work, particle size analysis was performed to characterize and determine the content of individual particle sizes of the ecological fillers, eggshells and coffee particles, as a percentage.

**Figure 1:**
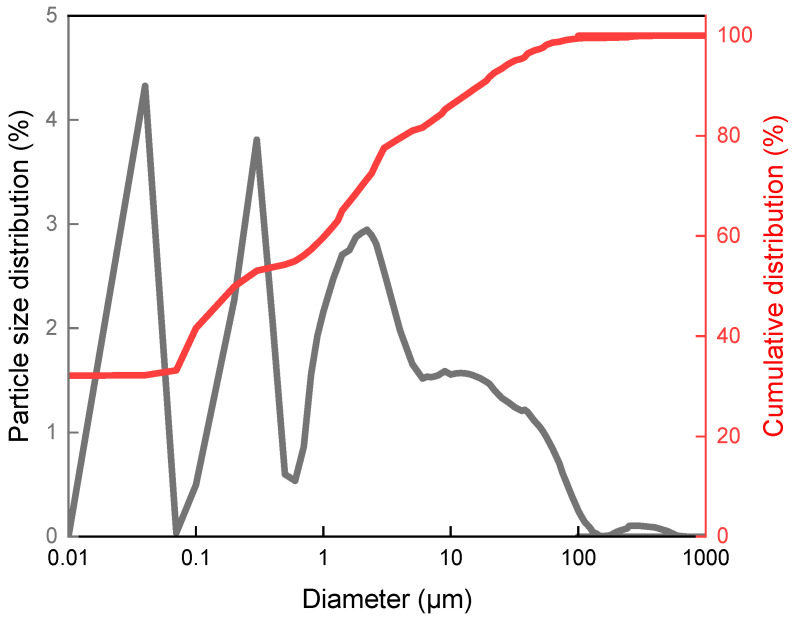
Correlation of particle diameter with particle size distribution and cumulative distribution for hen eggshells.

In Figure 1, the cumulative distribution and particle size distribution for hen eggshells are presented. The maximum particle diameter found in the tested material was 100 μm. Particles with a diameter of 1 [μm] constituted up to 60%, while those with a diameter of 0.01 μm constituted about 40%.

**Figure 2:**
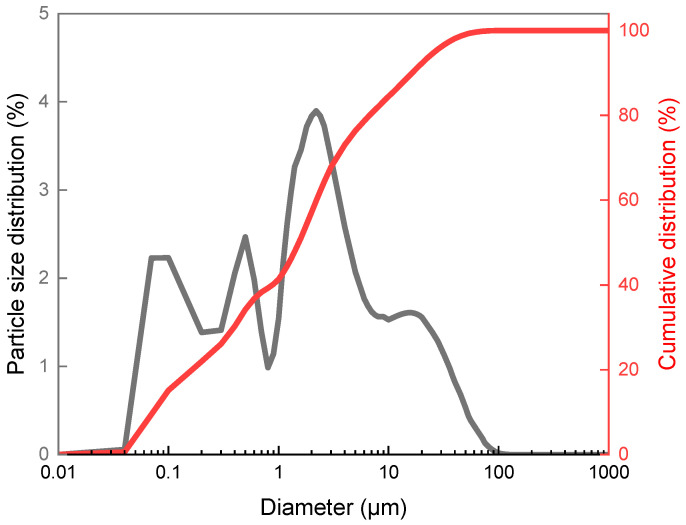
Correlation of particle diameter with particle size distribution and cumulative distribution for coffee.

In Figure 2, it can be seen that approximately 80% of the coffee powder analyzed by mass had a particle size of approximately 10 μm, while 40% of the volume consisted of particles up to 1 μm.

Due to the characteristics of the extrusion process, and after analyzing the above results, it was determined that the materials to be produced had to be made using a 0.6 mm head diameter. Attempts to produce composites using a 0.4 and 0.5 mm head diameter resulted in clogging.

To determine the composition of filler particles, XRF analysis of the chemical composition was performed for the polylactide–eggshell and polylactide–coffee ground composites.

Hen eggshells are primarily composed of calcium carbonate, phosphorus in the form of calcium phosphate and silicon (Table 3). XRF analysis revealed the presence of these elements, along with other elements that may act as contaminants or have a negligible effect on the chemical composition. It is worth noting that manufacturing the composite on a single-screw extruder creates the risk of structural inhomogeneity. This is due to the way that the polymer and filler are mixed. Based on the eggshell percentage calculation, these materials were mixed in a 90:10 ratio. In this composite, the chemical content of polylactide is 95.75%, which is lower than expected. This may also be due to the measurement method, which assumes that only the sample surface area is measured, with a maximum of 2 mm inside the test object.

Analysis of the chemical composition of the polylactide–coffee composite indicated the presence of silicon and potassium compounds in the tested sample (Table 4). It also revealed a lower-than-expected percentage of filler. In this case, the goal was to produce a composite composed of 90% polylactide and 10% coffee. It is worth noting that, as with the previous material, there are certain deviations. As already mentioned, this may be due to several factors including the mixing process, extrusion, and additive manufacturing, as well as the method used to measure the chemical composition.

### 2.2. Characterization and Method of Testing

Static tensile tests were performed on an MTS Criterion Model 43 hydraulic testing machine (MTS System Corp., Eden Prairie, MN, USA) with a measuring range of up to 30 kN using an axial extensometer. Stress relaxation was performed at a rate of 1 mm/min while maintaining a constant strain of 0.4 mm.

Images were taken using a JSM-IT200 InTouchScope scanning electron microscope (JEOL Ltd., Tokyo, Japan). To obtain appropriate conductivity during measurements, all samples were sputtered with gold.

The degree of crystallinity (*X_c_*) was calculated using the following Equation (1):

(1) $$X_{c}=\frac{∆H_{cc}}{∆H_{0}}\times 100\%$$

where

$∆H_{cc}$ is the specific melting enthalpy measured (J/g);

$∆H_{0}$ is the theoretical 100% crystalline polymer melting enthalpy, which equals 110 J/g for PLA [37].

## 3. Results

### 3.1. Mechanical Properties

Some of the basic parameters are strength and deformation during static and dynamic tests. To analyze the potential for the further use of the manufactured polylactide-based composites, static tensile and stress relaxation tests were performed. Based on the stress relaxation test results, a Zener model was calculated and graphically presented as a creep model.

**Figure 3:**
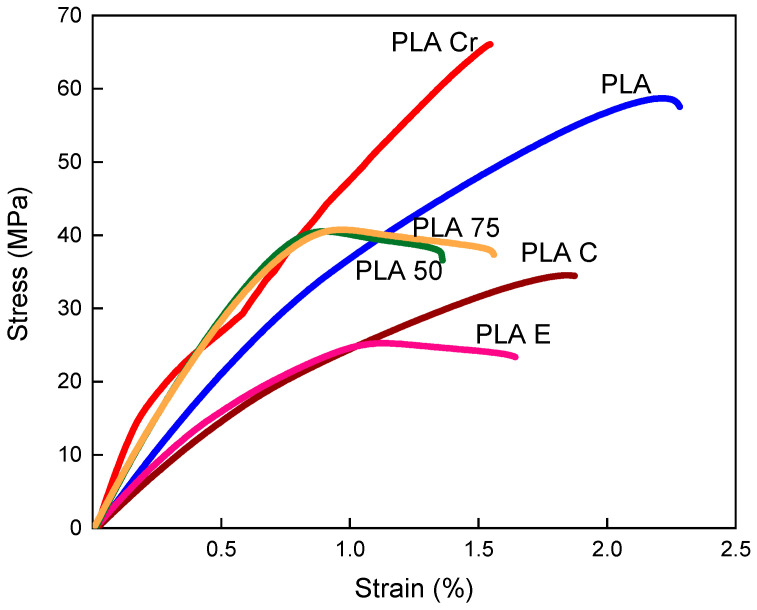
Stress–strain curves for polylactide and polylactide-based coffee grounds and eggshells composites.

In Figure 3, stress–strain curves for testes composites are presented. The most durable material tested was polylactide subjected to the crystallization process. These samples were characterized by a high Young’s modulus and high tensile strength. They had a strength of 66 [MPa], and there was no plastic range. The crystallization process fused the porous structure of FDM-printed samples, which increased the tensile strength. Polylactide made with 100% infill also has a high strength of 58 [MPa], but is characterized by a larger plasticity range, which means the sample is capable of significantly higher deformation under static loads. Reducing the degree of infill from 100 to 50 and 75% resulted in a significant decrease in strength properties to approximately 40 MPa, while the presence of voids, associated with the lack of infill, makes PLA 75 more prone to plastic deformation compared to PLA 50. The polylactide-based coffee composite has the highest content of the produced eco-friendly composites, at approximately 34 MPa. It is also characterized by the highest deformation of approximately 1.8%. The PLA-based composite with hen eggshells has a strength of approximately 25 MPa, which makes its bonds 22% weaker. The strain equals 1.4%, which also reduces the plastic deformation of this material by 23%. This is due to the presence of round coffee particles in the composite structure, which, according to fracture mechanics theory, are able to better transfer stress and dissipate mechanical energy during static loading. In the case of hen eggshells, stress concentration occurs at the corners of the particles. Their irregular shape and lack of dispersion in the structure reduce its mechanical properties. Increasing the symmetry and regularity of the distribution in the design of its internal structure would increase the possibility of achieving higher strength values.

### 3.2. Stress Relaxation

Stress relaxation tests were conducted to determine the rheological properties of polylactide prepared with varying degrees of filler content and the correlations between crystallized and non-crystallized materials. The study was conducted on polylactide samples prepared with 100%, 75%, and 50% filler, crystallized and enriched with organic fillers, eggshells and coffee grounds, at room temperature. Investigating this phenomenon can also contribute to conclusions regarding the material’s usefulness and the load-bearing capacity of potential structures or components that could be manufactured from it in the future.

**Figure 4:**
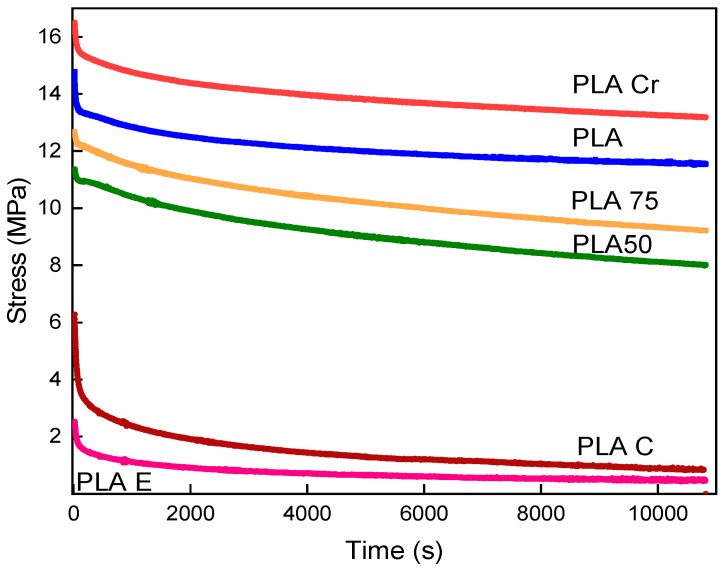
Stress relaxation curves of pure polylactide, after the crystallization process, with different degrees of infill and with ecological fillers: hen eggshell and coffee grounds.

Figure 4 shows the stress relaxation curves for pure PLA, crystallized PLA, PLA with different degrees of infill, and composites reinforced with coffee grounds and hen eggshells. After reaching the maximum strain value, the material exhibited typical behavior for this type of testing. Stresses rapidly decreased in the first few seconds, stabilizing over time and gradually decreasing. The highest stress relaxation was observed for polylactide subjected to the crystallization process. The maximum stress drop was approximately 17 [MPa]. The values obtained in the test correlate with the strength of the tested material. A lower degree of infill results in less of a capacity to withstand cyclic loads and permanent strain. PLA subjected to the crystallization process and without thermal treatment tended to exhibit higher force stabilization. The curves for samples made with incomplete infill were characterized by a major stress drop during the test. This was also related to the aforementioned strength of the material and the presence of voids in the sample. The voids increased the potential of microcracks, as well as propagation, which caused permanent deformation of the material. This is the consequence of faster decohesion compared to fully filled elements.

As for the strength properties of polylactide with eggshell and coffee grounds, the homogeneity of the structure influences stress relaxation. PLA with coffee particles exhibits a better ability to dissipate stress. This may be due to the presence of round particles within the matrix, which have a greater ability to dissipate energy. This shape enhances the material’s strength properties. The trend for this process is typical for the pure material. A rapid increase in stress values is visible until the required strain is achieved, followed by a rapid, momentary decrease, and then a slow oscillation towards lower values. As in the case of the static tensile test, the polylactide–eggshell composite demonstrated the lowest potential for mechanical energy dissipation. This is related to the irregular, angular shape of the powder particles within the matrix, which is detailed in the scanning electron microscope images.

### 3.3. Linear Standard Zener Model—Relaxation and Creep Analysis

**Figure 5:**
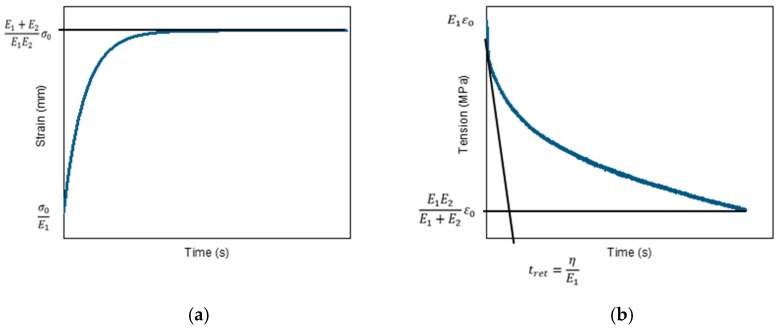
Graphical description of creep (**a**) and stress relaxation (**b**).

In order to determine the basic rheological properties of the produced materials, stress relaxation tests were performed. The general form of Zener’s model is illustrated in Figure 5, and the corresponding mathematical expression is given in Equation (2).

The standard linear model (the Zener model) is a classical approach to describing the behavior of viscoelastic materials. It combines an elastic element, E_1_, in series with parallel spring system, E_2_, and sticky η. The model allows for a description of both the stress relaxation and creep of the material. The derivation for the standard model leads to the following differential equation:

(2) $$\frac{\eta }{E_{1}+E_{2}}\cdot \dot{\sigma }+\sigma =\frac{{\eta \cdot E}_{1}}{E_{1}+E_{2}}\cdot \dot{\varepsilon }+\frac{E_{1}E_{2}}{E_{1}E_{2}}$$

where

η—Dynamic viscosity;

E_1_—Elastic modulus of the lower spring in the series branch [MPa];

E_2_—Elastic modulus of the parallel spring [MPa];

$\dot{\sigma }$—Time derivative of stress [MPa/s];

σ—Stress over time [MPa];

ε—Strain over time.

This equation relates stress σ and its time derivative $\dot{\sigma }$ with the strain ε and its derivative $\dot{\varepsilon }$. This model allows us to describe both phenomena:

(1) Stress relaxation for ε = const. derivative $\dot{\varepsilon }$ = 0, so the equation simplifies to Equation (2):

(3) $$\frac{\eta }{E_{1}+E_{2}}\cdot \dot{\sigma }+\sigma =\frac{E_{1}E_{2}}{E_{1}E_{2}}\cdot \varepsilon$$

Solving this equation leads to an exponential decrease in stress σ(t).

(2) Creep for σ = const. derivative $\dot{\sigma }$ = 0, so the equation simplifies to Equation (4):

(4) $$\sigma =\frac{{\eta E}_{1}}{E_{1}+E_{2}}\cdot \dot{\varepsilon }+\frac{E_{1}E_{2}}{E_{1}+E_{2}}\cdot \varepsilon$$

where

$\dot{\varepsilon }$—Time derivative of strain [1/s].

The solution leads to an increase in strain ε(t) over time, which describes the creep behavior of the material. The Zener model allows for a realistic description of the PLA behavior over time—both in terms of stress relaxation and creep. Unlike simple models, it accounts for both the immediate elastic effect and the delayed viscous response of the material.

**Figure 6:**
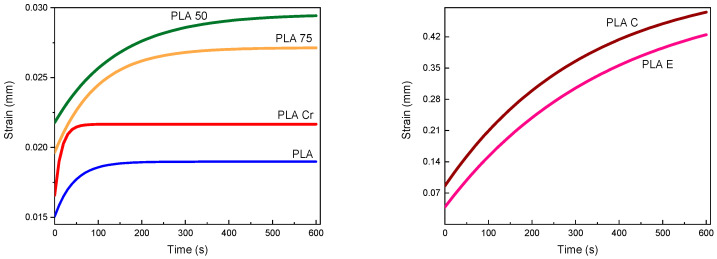
Creep curve based on the Zener model.

Based on the described dependence between creep and stress relaxation in the Zener model, calculations were performed, the results of which are presented in Figure 6. Each of the material groups presented is characterized by an initial steep increase in strain over time, caused by the occurrence of instantaneous elasticity. This increase then slows down, and the asymptotic curve approaches a limiting value, which is associated with steady state strain.

According to the data in Table 5, the most creep resistant material is pure polylactide, which has an amorphous structure. This structure is due to the presence of amorphous regions, which are associated with chains that move easily during deformation, and crystalline regions. They are ordered and strongly interconnected. Crystallized polylactide is characterized by the presence of only crystalline structures, which hinder the movement of polymer chains, resulting in a reduced tendency to creep. The increased modulus of elasticity makes the material less susceptible to viscoelastic deformation. Furthermore, the crystalline phase is characterized by a higher softening temperature, which reduces the possibility of deformation under a given load.

Polylactide with 75% and 50% of infill is characterized by higher creep potential because the material’s structure is designed in a lattice-like manner. Under load, energy is dissipated in the voids instead of being transferred to the polymer chains. The voids dissipate energy in the direction of the load. A lower infill level causes more voids that increasingly fuse together during deformation.

Composites with coffee particles and eggshells in a polylactide matrix exhibit a similar tendency to creep. This may be due to the presence of smaller particles compared to eggshells and their more even distribution in the matrix, which results in higher creep. PLA E starts with a higher initial strain, suggesting that it is more susceptible to immediate deformation under load. CaCO3 particles have weaker adhesion to the PLA matrix, which could have contributed to the formation of micro-defects, voids, and weaker bonds, resulting in a higher initial strain and a higher final creep rate. The fibrous coffee particles may have partially stabilized the structure, which is why the initial strain is lower and the curve increases smoothly.

This shows that in the case of polymer composites, not only the type of filler but also its quality, particle size, processing and adhesion to the matrix determine the creep potential.

### 3.4. Thermal Analysis

Differential scanning calorimetry measurements were performed to determine the thermal properties of the composites. Based on this, the following results were presented in Table 6 with their characteristic temperature ranges and energies.

**Figure 7:**
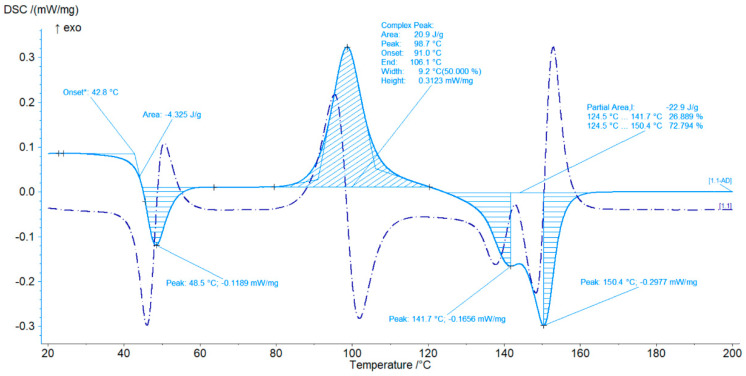
DSC curves of polylactide with 10% of coffee grounds. *—estimated start.

The DSC curve indicates that the coffee-based polylactide composite, manufactured using the FDM method, exhibits temperatures characteristic of PLA (Figure 7). The DSC curves of polylactide with 10% of coffee grounds are also shown in the figure. There is a noticeable increase in the cold crystallization energy for the resulting material, which increased by approximately 4%. The melting point begins at 124 °C. The first peak is visible at 141.7 °C. Two peaks are present, indicating the presence of an additional coffee phase. During the additive manufacturing of polylactide with 10% of coffee, it is possible to reduce the additive manufacturing temperature, which contributes to reducing process time, energy costs, carbon dioxide emissions, and the amount of polymer that can contribute to environmental pollution.

A very distinct cold crystallization peak at 98.7 °C is also visible. This peak has a 34% greater surface area compared to that of pure polylactide. The resulting crystalline phase melts until it reaches a temperature of 150.4 °C. There is an increase in the peak energy occurring in the melting temperature region, which is 57% higher than that for PLA.

**Figure 8:**
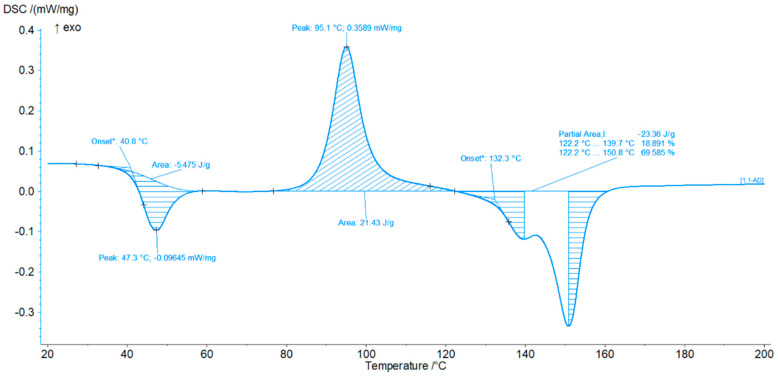
DSC curves of polylactide with 10% of eggshells. *—estimated start.

Differential scanning calorimetry studies revealed, similarly to the PLA-based egg composite, a double peak in the melting temperature range (Figure 8). The DSC curves of polylactide with 10% of eggshells are also shown in this figure. The second phase present in the material is coffee. The melting process begins at 122 °C, with the first peak occurring at 139 °C. The presence of polylactide is evident in the appearance of a second peak at 151 °C. The cold crystallization peak area also increased by 0.5 J/g. A characteristic feature of both composites is a significant increase in cold crystallization energy by 1 J/g compared to that of pure polylactide. The cold crystallization temperature also reaches a range that is lower by approximately 16 °C.

### 3.5. SEM Analysis

To analyze the phenomena occurring in composite materials produced using a 3D printer, scanning electron microscope images of the individual materials structures were prepared. The analysis focused on the impact of particles on the matrix of the produced composites, as well as their type and size for individual fillers. The appearance of particles added to the polylactide, according to the literature, was also considered in the published SEM images to correctly identify the fillers in the PLA matrix.

**Figure 9:**
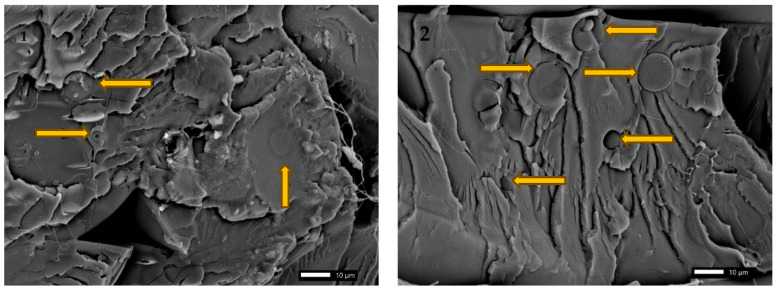
SEM analysis of polylactide with 10% of coffee grounds on the surface of a single layer at a 1000× magnification, (1) structure after static tensile test (2) individual coffee particles.

Figure 9 shows the microstructure of a polylactide composite with coffee particles. Their shape is very close to spherical, resulting in improved strength parameters for individual particles. Stress concentrations do not occur at the corners, so they are better able to dissipate mechanical energy than irregularly shaped particles. Additionally, the problem of particle adhesion between the matrix is visible. This can result in an increased likelihood of cracking in the composite produced with the coffee filler. The voids between the filler added to the polylactide caused a lack of structural homogeneity, which subsequently contributed to the increased hydrolytic degradation of the tested material. These properties will positively impact the carbon footprint and the environmental burden of polymer waste. The second photo also shows a microcrack formed within the coffee particle. It is also possible that it occurred during the additive manufacturing process of the composite or during operation. This means that it is worth taking a closer look at this phenomenon in the future. The parameters used in the additive manufacturing process can significantly influence the occurrence of this type of material discontinuity. This may also be related to the narrow temperature range within which the composite can be manufactured additively, as it ranged from 190 to 200 °C. Using a higher printing temperature involved initiating the burnout process in the 3D printer nozzle. The above photos also show a characteristic feature of polylactide: the presence of individual, drawn-out fibers on the material surface during the decohesion process.

**Figure 10:**
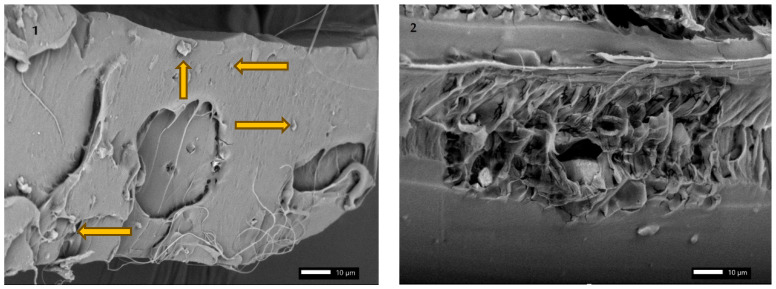
SEM analysis of polylactide with 10% of eggshells on the surface of single layer at 1000× magnification, (1) individual particles of eggshells (2) degree of homogeneity.

The structure of a composite of hen eggshells with a polylactide matrix was also analyzed using a scanning electron microscope (Figure 10). The figure shows the SEM analysis of polylactide with 10% of eggshells on the surface of a single layer at 1000× magnification. The particles in this material structure exhibit a wide variety of shapes. Sharp edges can also cause stress concentrations that will occur during cyclic and static loading. On the other hand, the dispersion of particles with sharp corners and arranged at various angles can increase strength properties. Using technology that allows for their conscious orientation could increase the possibility of mechanical energy dissipation in specific directions, which would result in the anisotropy of properties. As can be observed for the coffee ground composite with a polylactide matrix, there are noticeable areas where individual polylactide fibers formed during decohesion.

Microcracks are also visible in SEM images as a consequence of structural inhomogeneity. This phenomenon typically occurs in one direction, creating perpendicular, smaller cracks along the main stress path. Additionally, areas where the material has decohesed also indicate the formation of smaller microcracks on the surface of the polylactide-eggshell composite.

## 4. Conclusions and Future Development

Analysis of the physical and mechanical properties obtained in this study showed that the polylactide-based coffee composite had the highest strength, at approximately 34 MPa, and a strain of 1.8%. Properties deteriorated in the case of the polylactide filled with eggshell particles. The strength decreased by 9 MPa, and the strain decreased by 1.4%. This may be due to the shape of the particles used to produce the materials. SEM images demonstrated that the presence of round coffee particles can better transfer stress and dissipate mechanical energy. In the case of the eggshell composite, stress concentration occurs in the corners, which may lead to premature crack degradation.

A significant change in thermal properties during differential scanning calorimetry measurements is the shift in melting point and the presence of an additional coffee and hen eggshell phase. The melting point of the first composite material begins at 124 °C. It is possible to reduce the manufacturing temperature, electricity costs, carbon dioxide emissions, and process time. The cold crystallization temperature is approximately 16% lower compared to pure polylactide.

Based on the research conducted, it can be concluded that stress relaxation is dependent on the material strength. A higher composite materials tensile strength leads to higher potential for stress relaxation. Crystallinity reduces the potential for creep, while the addition of organic fillers increases the tendency for this process. This is related to the stiffness of the chains present in the polymer structure. It is worth checking the biocompatibility of these polymers, because the creep phenomenon can be used in implants for the human body, which require a certain degradation time.

## Figures and Tables

**Table 1:** Three-dimensional printing parameters.

Parameter	Value
Nozzle temperature [°C]	190
Bed temperature	50
Print angle [°]	45
Nozzle size [mm]	0.6
Degree of infill [%]	100
Speed print [mm/s]	45
Layer hight [mm]	0.1
Fan efficiency [%]	20

**Table 2:** Characterization of samples including chemical composition.

Sample Characterization	Composition
PLA	Pure polylactide with 100% degree of infill
PLA Cr	Pure polylactide with 100% degree of infill after crystalization process
PLA 75	Pure polylactide with 75% degree of infill
PLA 50	Pure polylactide with 50% degree of infill
PLA C	Polylactide with 10% of coffee grounds
PLA E	Polylactide with 10% of hen egg shells

**Table 3:** Chemical composition analysis of polylactide with 10% of eggshells.

Analyte	Contents [%]
Ca	2.656
Si	0.669
P	0.426
S	0.122
Cl	0.051
K	0.038
Ti	0.224
Fe	0.013
Pb	0.013
Bi	0.010
Zn	0.010
Tl	0.010
Sr	0.006
Br	0.003
C_3_H_6_OH	95.750

**Table 4:** Chemical composition analysis of polylactide with 10% of coffee grounds.

Analyte	Contents [%]
Si	2.004
K	1.235
Cl	0.281
Ca	0.230
Si	0.220
Ti	0.060
Pb	0.050
Fe	0.050
Hg	0.030
Tl	0.030
C_3_H_6_OH	95.812

**Table 5:** Creep comparison table for individual eco-friendly composites.

Specification	Start (t = 0)	End (t = 600)	Creep (Δε)
PLA	0.0151	0.0190	0.0039
PLA Cr	0.0166	0.0217	0.0051
PLA 75	0.0196	0.0271	0.0075
PLA 50	0.0218	0.0294	0.0076
PLA C	0.0384	0.4246	0.3862
PLA E	0.0858	0.4751	0.3893

**Table 6:** DSC results of pure PLA and composite ecological materials.

Material	Tg (°C)	Tcc (°C)	ΔHcc (J/g)	Tm (°C)	ΔHm (J/g)	Xc (%)
PLA [37,38,39]	62.4	~122	10.4	151.8	19.23	8%
PLA C	~48.5	~98.7	~20.9	141–150 (mainly peak 150.4)	~22.9	~1.8%
PLA E	~47.3	~95.1	~21.4	139–151 (mainly peak 150.8)	~23.4	~1.8%

## Data Availability

The original contributions presented in this study are included in the article. Further inquiries can be directed to the corresponding author.
